# Effect of dynamic cerebral autoregulation of cerebral blood flow regulation during cold pressor test

**DOI:** 10.14814/phy2.70813

**Published:** 2026-03-08

**Authors:** Shigehiko Ogoh, Kanoko Ito, Megumi Kaji, Narumi Kunimatsu, Kinu Tatsuta, Manabu Shibasaki

**Affiliations:** ^1^ Department of Biomedical Engineering Toyo University Saitama Japan; ^2^ Neurovascular Research Laboratory, Faculty of Life Sciences and Education University of South Wales Glamorgan UK; ^3^ Endowed Laboratory from the Sumitomo Electric Group CSR Foundation Nara Women's University Nara Japan; ^4^ Graduate School of Humanities and Sciences Nara Women's University Nara Japan; ^5^ Faculty of Engineering Nara Women's University Nara Japan

**Keywords:** cerebral autoregulation, cold pressor reflex, middle cerebral artery, vasoconstricton

## Abstract

The cold pressor test (CPT) is a clinical stress test commonly used to evoke acute pressor and respiratory responses, accompanied by increased sympathetic nerve activity. Cerebral blood flow (CBF) is well‐preserved during CPT despite elevated arterial blood pressure (ABP), but the underlying mechanisms remain unclear. We hypothesized that dynamic cerebral autoregulation (dCA) contributes to this CBF preservation, preventing overperfusion under stress‐induced hypertension. Thirty‐seven healthy volunteers performed CPT for 3 min, while ABP, middle cerebral artery mean velocity (MCA V_mean_), and end‐tidal carbon dioxide partial pressure (P_ET_CO_2_) were measured. CPT significantly increased ABP (*p* < 0.001) and decreased P_ET_CO_2_ (*p* = 0.032), whereas MCA V_mean_ remained unchanged (*p* = 0.976), resulting in a significant decrease in cerebrovascular conductance index (CVCi) (*p* < 0.001). The CPT‐induced relative change in CVCi was significantly associated with the low‐frequency (LF) phase (*p* < 0.001). Notably, the CPT‐induced decrease in P_ET_CO_2_ was significantly associated with the decreases in MCA V_mean_ (*p* < 0.001) and in %CVCi (*p* = 0.009). These findings suggest that dynamic cerebral autoregulation, as well as the respiratory response (change in P_ET_CO_2_), plays a key role in maintaining CBF during CPT‐induced elevations in ABP. Furthermore, individual differences in dCA and respiratory responses may account for variability in cerebrovascular responses to acute pressor stimuli.

## 1 INTRODUCTION

The cold pressor test (CPT) has been widely used as an experimental model to evoke sympathetic activation and pressor responses in humans. Previous studies have demonstrated that CPT induces changes in cerebral haemodynamics, suggesting active cerebrovascular regulation in response to acute sympathetic stress (Micieli et al., 1994; Roatta et al., 1998). However, the mechanisms underlying these responses remain incompletely understood, as cerebral blood flow is tightly regulated by both autoregulatory processes and arterial CO_2_ tension. It is applied to evaluate the effects of elevated sympathetic activation or cardiovascular stress on the cardiovascular system and to clinically identify the risk of cardiovascular disease (Lovallo, 1975; Rubenfire et al., 2000; Silverthorn & Michael, 2013; van Mil et al., 2017). For instance, the evaluation of common carotid artery (CCA) responsiveness to CPT has recently emerged as a surrogate marker of coronary artery responsiveness, and loss of this response has been associated with an increased risk of heart disease (Rubenfire et al., 2000; van Mil et al., 2017). The CPT has also been used to assess cerebrovascular responsiveness, most commonly by measuring changes in cerebral blood flow (CBF) velocity using transcranial Doppler. However, findings vary across studies in neurologically intact healthy individuals; some report no change in CBFV (AlSalahi et al., 2020; Castellani et al., 2006; Vianna et al., 2012; Washio et al., 2020), while others report an increase (Fluck et al., 2017; Zvan et al., 1998). Thus, the findings are inconsistent, and the underlying mechanisms remain unclear.

Our recent study (Washio et al., 2020) demonstrated that CBF velocity was well preserved during CPT at both MCA and PCA as well as no change in dynamic cerebral autoregulation (dCA) despite elevated ABP. Although the underlying mechanisms remain unclear, as mentioned previously, cerebrovascular control of CBF is influenced by multiple, potentially overlapping and redundant regulatory pathways, including autoregulation, SNA and cerebrovascular reactivity to carbon dioxide (CO_2_) tension (Willie et al., 2014). We speculate that the maintenance of CBF despite elevated ABP is likely due to dCA; however, direct evidence for this is lacking. Other studies (Allison et al., 2024; Toschi‐Dias et al., 2024) have suggested that CPT‐induced changes in CO_2_ and SNA may affect CBF responses during the CPT. However, these factors remain controversial, as no change in CBF has been observed even during CPT in the absence of end‐tidal partial pressure of CO_2_ (P_ET_CO_2_) changes (Washio et al., 2020). One important study (Toschi‐Dias et al., 2024) investigated the effect of SNA on CBF during CPT using transfer function analysis (TFA) coherence. While this method can estimate the influence of SNA on CBF, it cannot quantify the degree of contribution (e.g., even a small contribution may appear as a strong influence), and thus it cannot determine the specific role of SNA in regulating CBF during the large step increases in ABP induced by CPT. Their findings suggest that SNA may influence CBF responses during CPT; however, it remains unclear whether this effect contributes to the maintenance of CBF despite elevated ABP during CPT. Additionally, it is noteworthy that, in patients with spinal cord injury (SCI), CBF responses during CPT are not significantly different from those observed in healthy individuals (Sarafis et al., 2022; van der Scheer et al., 2018), suggesting that the role of SNA in regulating CBF during CPT may be limited. Therefore, further investigations are necessary to elucidate the mechanisms by which CBF is preserved despite elevated ABP during CPT.

Nevertheless, understanding this regulation is important for evaluating CBF control under conditions of elevated SNA or ABP. The CPT may serve as a useful tool to identify cerebrovascular disease risk, similar to its established utility in assessing cardiac disease risk. In the present study, to investigate the mechanisms underlying the maintenance of CBF during CPT despite elevated ABP, we hypothesized that dynamic cerebral autoregulation contributes to this CBF preservation. We further speculated that dynamic cerebral autoregulation remains effective during CPT, as our previous study demonstrated that it is well preserved in both anterior and posterior cerebral arteries under CPT conditions (Washio et al., 2020). To test this hypothesis, we assessed dCA at rest and changes in cerebral vascular conductance during CPT. In particular, we analyzed the association between individual differences in CBF responses and dCA as assessed by TFA, which should reflect the role of dCA in modulating the CBF response.

## 2 METHODS

### 2.1 Participants

Thirty‐seven healthy young volunteers (18 males and 19 females) participated in this study. The average age, body mass, and height were 22 ± 3 years, 59 ± 11 kg, and 165 ± 8 cm, respectively. Each subject provided written, informed consent after all potential risks and procedures were explained. All experimental procedures and protocols conformed to the Declaration of Helsinki and were approved by the Human Subjects Committee of Nara Women's University (# 23‐03). The subjects were free of any known cardiovascular and pulmonary disorders and were not using any prescribed or over the counter medications. Subjects were requested to abstain from caffeinated beverages for 12 h and strenuous physical activity and alcohol for at least 24 h before the day of the experiment.

### 2.2 Experimental protocol

Experiments were conducted in a temperature‐controlled laboratory (25°C ± 1°C). Following an explanation of the experimental procedure and the provision of written informed consent, participants rested in a supine position on a bed for approximately 30 min, during which the measurement devices were attached. Subsequently, 5 min of steady‐state data were obtained for the analysis of dCA, followed by the cold pressor test (CPT). For the CPT test, participants immersed their right hand in ice water (0°C–4°C) for 3 min.

### 2.3 Measurements

Middle cerebral artery (MCA) mean blood velocity (V_mean_) was measured by transcranial Doppler ultrasonography (WAKI; Atys Medical, St Genislaval, France) using a 2 MHz Doppler probe placed at the left temporal window. Heart rate (HR) was obtained from an electrocardiogram (Biomulti 1000, NEC, Tokyo, Japan). Beat‐to‐beat arterial blood pressure from a finger cuff was recorded from a finger using the Penaz method (Portapress, FMS, Amsterdam, The Netherlands), and intermittent arterial blood pressure was obtained by auscultation of the brachial artery via electrosphygmomanometry (STBP‐780, Colin, Tokyo, Japan) for 15 subjects. Left radial arterial waveforms were recorded using an applanation tonometry‐based automated blood pressure measurement device (EP608, Omron Colin, Kyoto, Japan), and electrocardiograms were simultaneously obtained with the electrocardiograph incorporated in this device for 22 participants (8 males and 14 females). Minute ventilation (VE) and end‐tidal partial pressures of carbon dioxide (P_ET_CO_2_) were sampled via a leak‐free mask, with gas partial pressures measured with a gas analyzer (ARCO2000‐MET; Arcosystem, Chiba, Japan). All measurements were performed in all participants except for the gas measurements, which were conducted in the 22 participants. These continuous variables were sampled at 20 Hz via a data acquisition system (MP150, BIOPAC Systems, Santa Barbara, CA, USA).

### 2.4 Data analysis

TFA assessed dCA during the steady‐state condition. Mean arterial blood pressure (MAP) and MCA V_mean_ were calculated across each cardiac cycle, interpolated with a third‐order spline, and resampled at 10 Hz for the TFA. Data analysis and interpretation were conducted according to established guidelines published by the International Cerebral Autoregulation Research Network (Claassen et al., 2016), including the removal of negative values for phase indicative of “wrap‐around” artifacts for frequencies <0.1 Hz. The transfer function between MAP and MCA V_mean_ was calculated in the very low frequency (VLF; 0.02–0.07 Hz), low frequency (LF; 0.07–0.20 Hz), and high frequency (HF; 0.20–0.50 Hz) ranges. TFA in the low‐frequency range of 0.07–0.20 Hz models short‐term regulation of CBF in response to changes in arterial pressure. Transfer function gain and phase shift reflect the relative amplitude and time relationships, respectively, between the changes in perfusion pressure and blood flow over the assessed frequency range. An increase in gain indicates a greater influence of MAP on CBF, and thus reflects an impairment in dCA. Phase shift was also considered as a criterion for evaluating dCA, where a decrease in phase reflects a more pressure‐passive relationship between MAP and MCA V_mean_ and is associated with a reduction in dCA (Panerai, 2008). The squared coherence function reflects the function of CBF power that can be related linearly to the MAP power within each frequency. Similar to a correlation coefficient, it varies between 0 and 1 and reflects the strength of the linear relationship between two values. The cerebrovascular conductance index of the MCA (CVCi) was calculated by dividing the MCA V_mean_ by the MAP.

### 2.5 Statistical analysis

All values are expressed as mean ± SD after normality was confirmed using the Shapiro–Wilk test. Comparisons between control and CPT conditions were analyzed using a paired *t*‐test (Statistica 7.0; StatSoft). Pearson correlation analysis was performed to assess the relationships between changes in P_ET_CO_2_ and responses of MCA V_mean_ and CVCi during the cold pressor test, as well as between TFA parameters and the relative change in CVCi. A P‐value of less than 0.05 was considered statistically significant.

## 3 RESULTS

**FIGURE 1:**
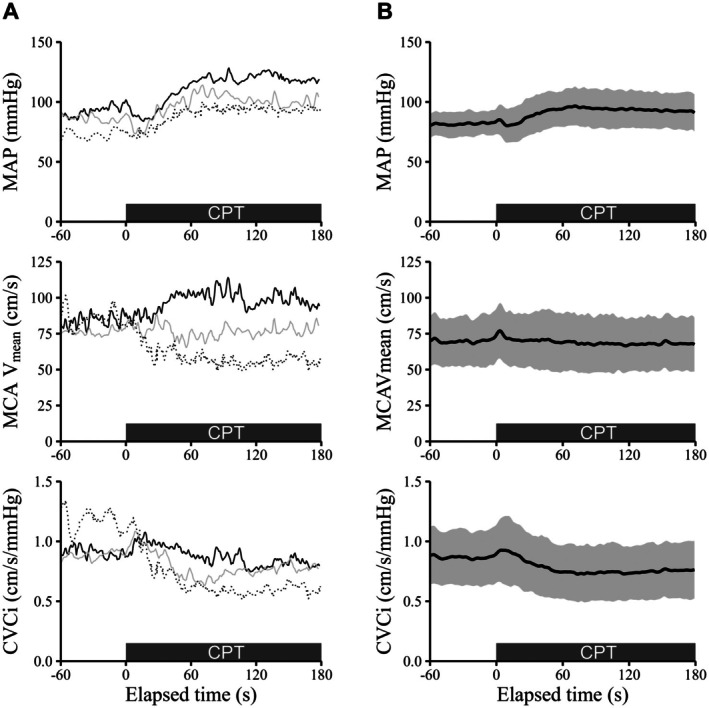
Time‐course changes in arterial pressure and cerebral blood flow during the cold pressor test (CPT). Panel A shows representative individual responses from three participants illustrating distinct middle cerebral artery mean velocity (MCA V_mean_) patterns during CPT: Minimal change, increase, and decrease. Among the 37 participants, 20 exhibited minimal changes in MCA Vmean (defined as <10% change), eight showed an increase, and nine showed a decrease during CPT. Panel B presents group‐averaged changes in mean arterial pressure (MAP), MCA V_*mean*_, and cerebrovascular conductance index (CVCi) expressed as mean ± standard deviation. CPT was applied from 0 to 180 s, as indicated by the shaded area. All variables are presented as absolute values. These data demonstrate marked inter‐individual variability in MCAV responses during CPT, while showing that MCA V_mean_ remained relatively stable at the group level despite a pronounced increase in MAP, accompanied by a reduction in CVCi.

**FIGURE 2:**
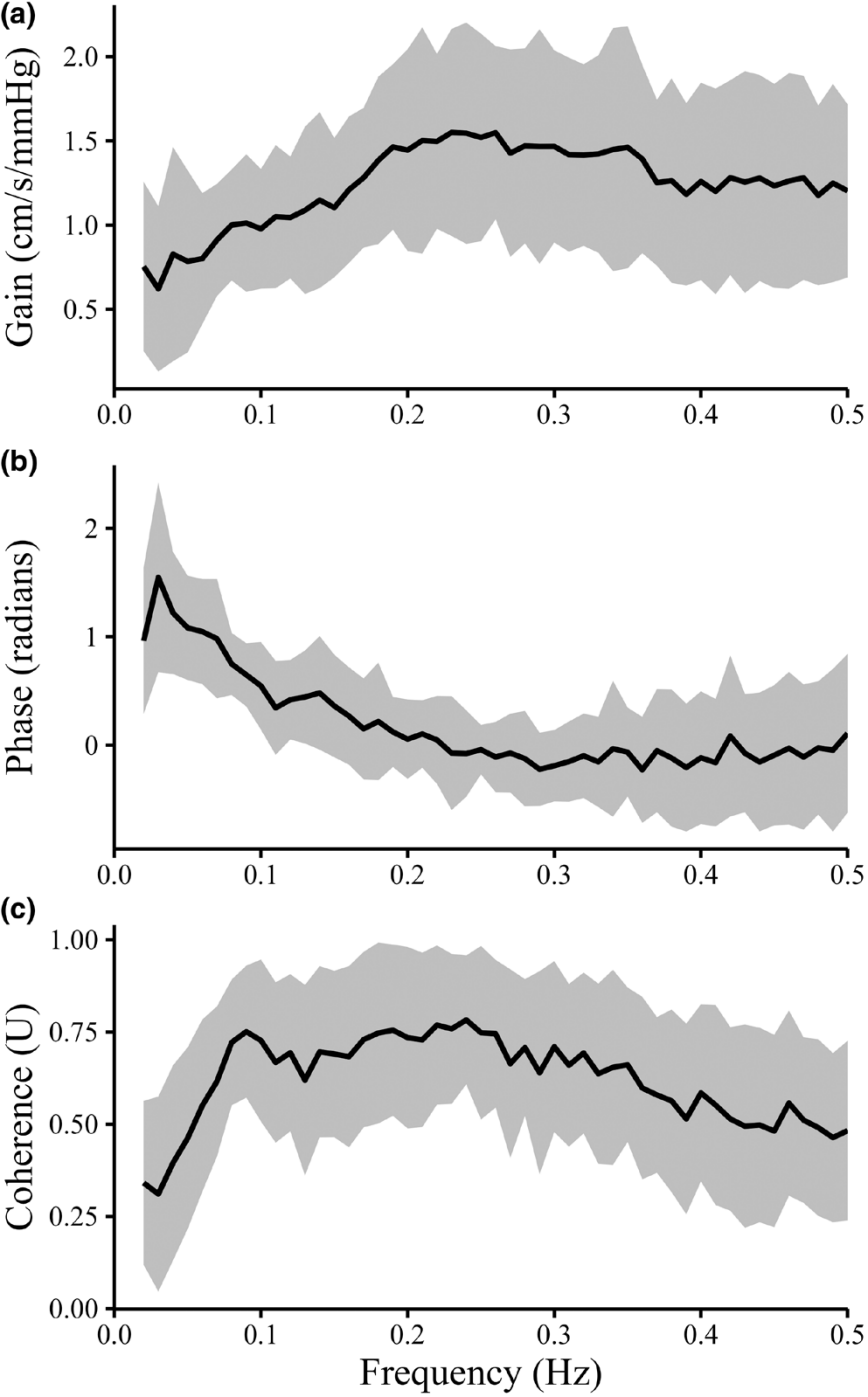
Group‐averaged transfer function analysis (TFA) results between beat‐by‐beat mean arterial pressure (MAP) and mean middle cerebral artery blood velocity (MCA V_mean_) at rest. Panels show frequency‐domain estimates of (a) gain, (b) phase, and (c) coherence across the very‐low‐frequency (VLF: 0.02–0.07 Hz), low‐frequency (LF: 0.07–0.20 Hz), and high‐frequency (HF: 0.20–0.50 Hz) ranges.

**FIGURE 3:**
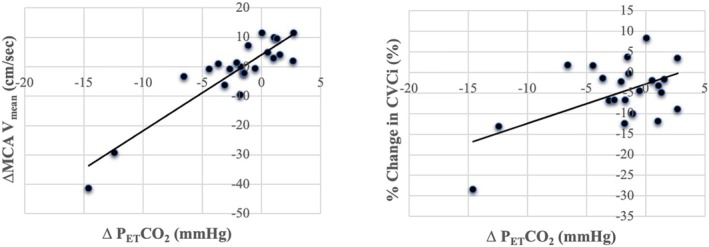
The correlation of end‐tidal carbon dioxide partial pressure (P_ET_CO_2_) with the percent change in middle cerebral artery mean blood velocity (MCA V_mean_, right panel) and cerebrovascular conductance index (CVCi, left panel) caused by cold pressure test (CPT).

**FIGURE 4:**
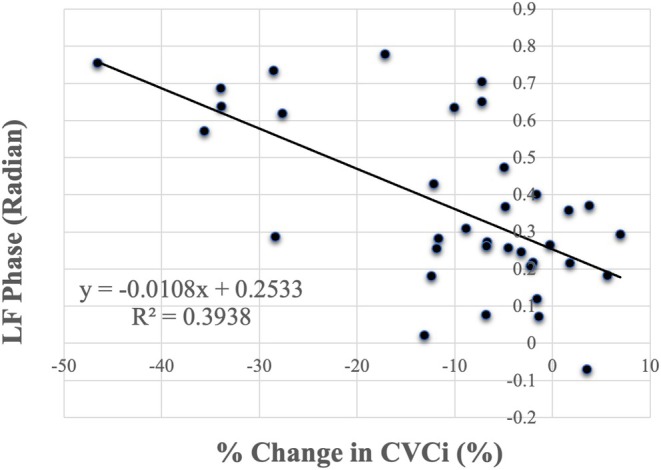
The relationship between the percent change in cerebrovascular conductance index (CVCi) caused by cold pressor test (CPT) and individual dynamic cerebral autoregulation (LF Phase).

CPT significantly increased HR and MAP due to sympathetic activation (HR: *p* = 0.008; MAP: *p* < 0.001; Table 1, Figure 1). Figure 2 shows the transfer function analysis (TFA) results describing the frequency‐dependent characteristics of dynamic cerebral autoregulation. However, MCA V_mean_ remained unchanged during CPT (*p* = 0.976). The MCA V_mean_ response to CPT was variable: while some participants showed a slight increase in MCA V_mean_ during CPT, the average response of MCA V_mean_ did not differ significantly between the control and CPT conditions. Consequently, CVCi significantly reduced during CPT compared with the control condition (*p* < 0.001). As shown in Figure 3, P_ET_CO_2_ decreased during the CPT due to hyperventilation (*p* = 0.032). The CPT‐induced changes in P_ET_CO_2_ were significantly associated with the CPT‐induced changes in MCA V_mean_ (*p* < 0.001) and in %CVCi (*p* = 0.009). However, this outcome appears to have been influenced by two subjects in Figure 3 who exhibited a marked decrease in P_ET_CO_2_. The dCA was evaluated using TFA between MAP and MCA V_mean_ only at rest (Table 2), as previous studies have demonstrated that CPT does not alter dCA. Interestingly, a significant relationship was observed between LF phase and the relative change (%) in CVCi (*p* < 0.001, Figure 4), indicating that stronger dCA was associated with a greater decrease in CVCi, demonstrating that CBF is well maintained despite a large increase in ABP.

**TABLE 1:** Hemodynamics at supine rest and during Cold Pressor Test (CPT).

	HR	MAP	MCA V_mean_	CVCi	P_ET_CO2
	(bpm)	(mmHg)	(cm/s)	(cm/s/mmHg)	(mmHg)
Control	65 ± 10	78 ± 11	69.2 ± 15.6	0.914 ± 0.256	40.0 ± 3.4
CPT	67 ± 10	90 ± 16	67.6 ± 18.3	0.774 ± 0.244	37.9 ± 4.2
Paired *t‐*test	*p* = 0.008	*p* < 0.001	*p* = 0.976	*p* < 0.001	*p* = 0.032

*Note*: Mean ± SD (*N* = 37 except for P_ET_CO_2_, *N* = 22).

Abbreviations: HR; heart rate; MAP, mean arterial pressure; MCA V_mean_, middle cerebral artery mean blood velocity; P_ET_CO_2_, partial pressure of end‐tidal carbon dioxide.

**TABLE 2:** Transfer function analysis (TFA) between middle cerebral artery blood velocity (MCA V_mean_) and mean arterial blood pressure (MAP) to assess dynamic cerebral autoregulation.

	Gain	(cm/s/mmHg)	0.784 ± 0.361
VLF	Phase	(radian)	1.138 ± 0.299
	Coherence	(U)	0.446 ± 0.157
LF	Gain	(cm/s/mmHg)	1.170 ± 0.313
	Phase	(radian)	0.370 ± 0.225
	Coherence	(U)	0.709 ± 0.131
HF	Gain	(cm/s/mmHg)	1.365 ± 0.405
	Phase	(radian)	−0.082 ± 0.175
	Coherence	(U)	0.615 ± 0.124

*Note*: Mean ± SD (*N* = 37).

Abbreviations: HF, high frequency (0.2–0.5 Hz); LF, low frequency (0.07–0.2 Hz); VLF, very low frequency (0.02–0.07 Hz).

## 4 DISCUSSION

In the present study, we investigated the mechanisms underlying the maintenance of CBF during the CPT, which induces significant elevations in ABP via sympathetic activation. Consistent with previous studies, CPT significantly increased ABP, while MCA V_*mean*_ remained unchanged on average, although some individuals showed an increase in CBF in response to CPT. Correspondingly, CVCi decreased significantly during CPT, indicating a compensatory vasoconstrictive response of the cerebral vasculature to elevated ABP. Importantly, a significant association was observed between the individual relative change in CVCi and resting dCA. These findings suggest that dCA plays a key role in maintaining CBF during acute pressor stress. In other words, assessing CBF responses during CPT may help identify individuals at risk of cerebrovascular disease or with impaired dCA.

Comparison with prior studies reveals some variability in findings regarding CBF responses to CPT. Some studies have reported increases in CBF (Fluck et al., 2017; Zvan et al., 1998), whereas others observed no significant changes (AlSalahi et al., 2020; Castellani et al., 2006; Vianna et al., 2012; Washio et al., 2020). These discrepancies may be attributable to differences in participant characteristics, measurement sites, or methodological approaches. For example, one study reporting an increase in CBF during CPT included middle‐aged individuals, whereas another study showing an increase used foot cold stimulation rather than hand immersion. Moreover, individual variability in CBF responses may differ between experimental groups. However, the reasons for these inconsistent findings regarding CBF responses to CPT remain unclear.

The temporal characteristics of cerebral blood flow velocity responses to sudden changes in arterial blood pressure have been well described, highlighting the importance of dynamic cerebral autoregulation in buffering acute pressor challenges (Panerai et al., 2001). In this context, dynamic cerebral autoregulation represents a key mechanism for stabilizing cerebral perfusion during transient elevations in arterial pressure. Our previous study (Washio et al., 2020) demonstrated that dCA remains effective during CPT. In that study, dCA was measured during CPT and found to be unchanged compared with the control condition. Based on these findings, dCA was not analyzed during CPT in the present study, as no differences between CPT and control conditions were expected. In addition, TFA requires at least 5 min of data (Claassen et al., 2016; Panerai et al., 2023), which is not feasible given the limited duration of CPT. Despite the absence of direct dCA measurements during CPT, the significant association observed between LF phase at rest and the relative change in CVCi further supports the notion that stronger dCA contributes to greater vasoconstrictive adjustments, thereby helping to preserve CBF under conditions of elevated ABP. Collectively, these findings suggest that dCA remains active during CPT and provide insight into inter‐individual variability in cerebrovascular responses. They highlight that individuals with more robust dCA exhibit better maintenance of CBF during acute hypertensive stress, thereby helping to protect the cerebral vasculature from overperfusion under these conditions. The present findings have important physiological implications. Maintaining CBF despite acute elevations in ABP is crucial for ensuring stable delivery of oxygen and nutrients to the brain. Our results suggest that individuals' differences in dCA may determine the extent to which CBF is preserved under sympathetic stress. Furthermore, the CPT may serve as a useful tool for evaluating cerebrovascular function and identifying individuals at risk of impaired cerebral autoregulation.

The decrease in P_ET_CO_2_ observed during the CPT may have contributed to the maintenance of CBF despite elevated ABP. In the present study, the CPT‐induced reduction in P_ET_CO_2_ was significantly associated with decreases in MCA V_*mean*_ and %CVCi (both, *p* < 0.01), indicating that ventilatory responses played an important role in modulating cerebral perfusion. Hyperventilation‐induced hypocapnia is a potent vasoconstrictor of the cerebral circulation and may therefore act as a protective mechanism against overperfusion during acute pressor stress. Importantly, hypocapnia has also been shown to augment dCA (Aaslid et al., 1989), suggesting a physiological interaction between respiratory control and autoregulatory mechanisms. Thus, the combined effects of reduced P_ET_CO_2_ and preserved dCA likely contributed to the effective regulation of CBF during CPT‐induced elevations in ABP.

Another possible mechanism influencing CBF regulation during CPT is sympathetic nerve activity (SNA). Although the cerebral circulation is richly innervated with sympathetic fibers, the influence of SNA on cerebral blood flow in humans is traditionally believed to be limited, particularly under resting conditions (Alm & Bill, 1973; Harper et al., 1972). This is because the cerebral blood flow is regulated differently from other peripheral vascular beds, given the relatively small vascular bed of the cerebral circulation and its strong regulation by cerebral autoregulation and arterial CO_2_ tension (Ogoh & Ainslie, 2009a, 2009b). More recent work has further emphasized that autonomic control of the cerebral vasculature differs fundamentally from that of peripheral circulations, with sympathetic influences remaining limited and highly context‐dependent even under conditions of physiological stress (Koep et al., 2022). In contrast, under hypertensive conditions, sympathoexcitation can directly induce cerebral vasoconstriction (Heistad et al., 1978; Patel et al., 2003; Rutland et al., 1980). Heistad et al. (1978) showed that sympathetic stimulation reduces CBF during severe hypertension in cats, dogs, and monkeys, despite minimal effects at rest. Similarly, Prazosin, an α₁‐adrenergic blocker, does not affect CBF at rest in normotensive humans (Patel et al., 2003) but increases CBF and lowers blood pressure in hypertensive patients (Rutland et al., 1980). Together, these studies (Heistad et al., 1978; Patel et al., 2003; Rutland et al., 1980) suggest that increased SNA limits cerebral arteriole dilation, thereby preventing regional overperfusion and disruption of the blood–brain barrier during marked elevations in arterial pressure. Accordingly, CPT‐induced increases in SNA may contribute to cerebral vasoconstriction and help prevent overperfusion during elevated ABP. However, evidence from recent human studies indicates that changes in sympathetic activity do not uniformly translate into alterations in cerebral blood flow. Although SNA has been suggested to influence CBF during CPT (Toschi‐Dias et al., 2024), studies in individuals with spinal cord injury show similar CBF responses to CPT as healthy participants (Sarafis et al., 2022; van der Scheer et al., 2018), indicating that the contribution of SNA to CBF regulation during CPT may be limited. Taken together, these findings highlight the complex and context‐dependent nature of sympathetic control of the cerebral circulation and underscore the ongoing controversy surrounding the role of SNA in cerebrovascular regulation (Brassard et al., 2017). In the context of the present findings, although SNA cannot be excluded as a contributing factor, our results suggest that it was unlikely to be the primary driver of CPT‐induced cerebral vasoconstriction. Instead, inter‐individual variability in ventilatory responses, leading to substantial reductions in P_ET_CO_2_ in some participants, appeared to exert a disproportionate influence on group‐averaged CBF responses. These observations indicate that CO_2_‐mediated cerebrovascular regulation, in conjunction with preserved dCA, represents a major determinant of CBF control during CPT, whereas sympathetic influences may play a secondary or modulatory role.

Several limitations should be considered. First, dCA was assessed using transfer function analysis only at resting conditions, and was not directly evaluated during CPT. Although previous work has demonstrated that CPT does not markedly alter dCA, the absence of direct measurements during CPT limits conclusions regarding moment‐to‐moment autoregulatory adjustments under acute pressor stress. Second, P_ET_CO_2_ measurements were obtained in a subset of participants (22 of 37), which constrained the statistical power for evaluating the relative contributions of ventilatory responses and dCA. Although significant associations were observed between CPT‐induced changes in P_ET_CO_2_ and both MCA V_*mean*_ and CVCi, these findings should be interpreted with caution and confirmed in larger cohorts with complete respiratory measurements. Third, although CPT is widely used as a model of sympathetic activation, we were unable to directly quantify SNA in the present study. Consequently, the independent contributions of SNA, CO_2_‐mediated effects, and other regulatory mechanisms, such as endothelial or myogenic responses, could not be fully isolated. Further studies are needed to confirm the contribution of P_ET_CO_2_. Finally, it should be noted that finger arterial pressure measurements obtained using volume‐clamp techniques, such as the Portapres system, may be susceptible to pronounced peripheral vasoconstriction during CPT. In several participants, excessive vasoconstriction at the finger likely compromised signal quality, resulting in unreliable or unrecordable pressure waveforms during the maneuver. Although these data were excluded from the analysis and additional validation was performed using brachial arterial pressure measurements, this limitation highlights an important methodological consideration for studies employing volume‐clamp methods under conditions of strong sympathetic vasoconstriction. Future investigations using CPT or similar stressors should carefully consider the potential influence of peripheral vasoconstriction on distal pressure measurements and, where possible, incorporate alternative measurement sites or strategies to mitigate this effect.

In conclusion, our findings indicate that dCA as well as respiratory response (change in P_ET_CO_2_) plays a key role in maintaining CBF during CPT‐induced elevations in ABP, and that individual differences in dCA and respiratory response may account for variability in cerebrovascular responses to acute pressor stimuli. These results underscore the importance of dCA and respiration for cerebrovascular homeostasis under conditions of sympathetic stress.

## AUTHOR CONTRIBUTIONS

The present study was conducted at Nara Women's University. The study was designed by Manabu Shibasaki and Shigehiko Ogoh, and performed by Megumi Kaji, Narumi Kunimatsu, Kinu Tatsuta, Kanoko Ito, and Manabu Shibasaki. The collected data were analyzed and interpreted by Shigehiko Ogoh, Kanoko Ito, and Manabu Shibasaki. This manuscript was drafted by Shigehiko Ogoh. All authors have approved the final manuscript submitted for publication and agree to be accountable for all aspects of the work and its interpretations.

## FUNDING INFORMATION

This study is supported by the Sumitomo Electric Group CSR Foundation, and partly supported by a Grant‐in‐Aid for Scientific Research [grant number 23K24727] from the Japanese Ministry of Education, Culture, Sports, Science and Technology.

## CONFLICT OF INTEREST STATEMENT

The authors declare no conflicts of interest.

## Data Availability

All data supporting the results are presented in the manuscript.
